# Adaptation of the Direct Assessment of Functional Status (DAFS):a new tool to assess functional changes in people with Down syndrome

**DOI:** 10.1590/1980-5764-DN-2025-0415

**Published:** 2026-07-17

**Authors:** Alexandra Martini de Oliveira, Carolina Lopes Rodrigues Vanetti, Fabiana Alencar, Aline de Souza Gonçalves Gomes da Conceição, Luana Dongue Martinez, Lívea Carla Fidalgo Garcez Sant´Ana, Claudia Lopes Carvalho, Guilherme Prado Mattar, Vinícius Leduc, Ana Paula Assunção Cecilio, Vitor Nascimento de Almeida, Maria de Fátima Rebouças da Silva, Octávio Ribeiro Gonçalves, Bruna Luiza Roim Varotto, Larissa da Silva Serelli, Elizabeth Prado Prestes Barra Teixeira, Orestes Vicente Forlenza

**Affiliations:** 1 Universidade de São Paulo, Faculdade de Medicina, Hospital das Clínicas, Departamento e Instituto de Psiquiatria, Laboratório de Neurociências, LIM27 – São Paulo SP, Brazil.; 2 Universidade Federal da Bahia, Faculdade de Medicina, Departamento de Saúde da Família, Curso de Terapia Ocupacional – Salvador BA, Brazil.

**Keywords:** Functional Status, Disability Evaluation, Trisomy 21, Activities of Daily Living, Estado Funcional, Avaliação de Deficiência, Trissomia do Cromossomo 21, Atividades Cotidianas

## Abstract

Functional decline in activities of daily living (ADL) is considered a marker of ageing and Alzheimer’s disease. However, there is a lack of performance-based instruments specifically designed to assess ADL in adults and older adults with Down syndrome.

**Objective:**

To describe the adaptation process of the Direct Assessment of Functional Status (DAFS) to assess the functional capacity of adults with Down syndrome.

**Methods:**

The Direct Assessment of Functional Status-Brazilian Version (DAFS-BR) was administered to 15 adults with Down syndrome (nine men and six women) who were divided into two diagnostic groups: stable cognition and suspected dementia or cognitive impairment. The process was conducted in two phases: phase one was characterized by an adaptation in the tasks. In phase two, (cultural and semantic) equivalences were verified, as well as structural aspects, including layout and instructions. This phase was essential for verifying the applicability and comprehensibility of newly adapted tasks

**Results:**

The DAFS-BR was adapted for the time orientation, communication (telephone use), moneyhandling skills, and shopping skills domains, considering the target population.

**Conclusion:**

The adaptation process of the DAFS-BR for people with Down syndrome was made considering linguistic, psychological, and cultural idiosyncrasies in the target population, with the input of experts with relevant experience in each domain. After psychometric studies, the Direct Assessment of Functional Status-Down Syndrome (DAFS-DS) could be considered the first ecological instrument for evaluating functional status in adults with Down syndrome in Brazil to enhance both clinical practice and research.

## RESUMO

O declínio funcional nas atividades da vida diária (AVD) é considerado um marcador do envelhecimento e da doença de Alzheimer. No entanto, há falta de instrumentos de avaliação baseados em desempenho especificamente desenhados para avaliar a AVD em adultos e idosos com síndrome de Down (SD).

**Objetivo:**

Descrever a adaptação da Avaliação do Estado Funcional (DAFS) para avaliar a capacidade funcional de adultos com síndrome de Down (SD).

**Métodos:**

A DAFS-BR foi aplicada em 15 adultos com SD (nove homens e seis mulheres) divididos em dois grupos diagnósticos: cognição estável e suspeita de demência ou comprometimento cognitivo. O processo foi conduzido em duas etapas: na primeira, foram realizadas adaptações nas tarefas e, na segunda, foram verificadas equivalências (culturais e semânticas), aspectos estruturais, incluindo layout e instruções. Esta etapa foi essencial para verificar a aplicabilidade e a compreensibilidade das tarefas recém-adaptadas.

**Resultados:**

A DAFS-BR foi adaptada nos domínios de orientação temporal, comunicação (uso do telefone), capacidade de gestão financeira e habilidades de compras, considerando a população alvo.

**Conclusão:**

O processo de adaptação da DAFS-BR para SD foi realizado considerando-se as particularidades linguísticas, psicológicas e culturais da população alvo, com a participação de especialistas com experiência relevante na área e em cada domínio. Após estudos psicométricos, o DAFS-SD poderá ser considerado o primeiro instrumento ecológico para a avaliação do estado funcional em adultos com síndrome de Down no Brasil, visando aprimorar tanto a prática clínica quanto a pesquisa.

## INTRODUCTION

 The term activities of daily living (ADLs) describes essential skills required to independently care for oneself, such as eating, bathing, and mobility. Importantly, ADLs are used as an indicator of a person’s functional status, who can perform ADLs, contributing significantly to overall quality of life^1^. People with Down Syndrome (DS), in turn, have cognitive features that may undermine their potential to perform ADLs, although the level of ADL impairment may vary significantly across that population (e.g., concerning independence or the ability to complete self-care tasks). 

 In addition to the cognitive and functional changes inherent to the syndrome itself, DS is also associated with a high risk of developing Alzheimer’s disease (AD)^2,3^. The risk of dementia in individuals with DS can be explained by biological aspects, in part, by the extra copy of chromosome 21, which is linked to the overexpression of certain proteins related to neurodegeneration, leading to deregulation of the amyloid precursor protein, as well as other proteins in the brain associated with increased oxidative stress and neurodegeneration^ 3-5^. Clinical studies have found that over 80% of DS adults aged 65 years or older show symptoms of Alzheimer-type dementia^6, 7^. This aspect is considered highly important, given that functional impairment is one of the main symptoms of AD, as often measured via an increasingly compromised ability to perform ADLs^8^. For instance, according to Listwan et al.^9^, there is clear evidence that functional decline accompanies cognitive decline in DS in a similar fashion as that observed in elderly adults without DS. 

 In that vein, assessment tools are key to detecting functional changes in the context of dementia, even in the DS population^10^. However, several functional assessments to evaluate ADL were developed for the general population. Yet, such assessments rely on a certain level of cognitive function and self-awareness to work; otherwise, individuals may not be able to accurately report their own functional capacities. Thus, whenever self-awareness is compromised, direct functional assessments (when information is obtained more ecologically by directly examining functional performance during ADL simulations) might provide a better fit^11^. Notably, one example is the Direct Assessment of Functional Status Scale (DAFS)^12^. 

 The DAFS consists of seven distinct dimensions: time orientation (eight items); communication skills (17 items); transportation (13 items); money-handling skills (21 items); shopping skills (eight items); eating (five items); and dressing and personal hygiene skills (13 items). Patients with cognitive impairment took about 30 to 35 minutes to complete the DAFS for validation^11,12^. Test-retest reliability over three to seven weeks indicated a high degree of stability over time. Correlation patterns between the DAFS and other measures of functional status confirmed the scale’s discriminant and convergent validity^11^. Importantly, this scale has been translated into Brazilian Portuguese and culturally adapted by our group, yielding the Direct Assessment of Functional Status-Brazilian Version (DAFS-BR)^11^. The Brazilian version has a score ranging from 0 to 105, with higher scores indicating superior performance. The domains evaluated are time orientation (16 points); communication skills (14 points); money-handling skills (32 points); shopping skills (20 points); dressing skills (13 points); and eating (10 points). This Brazilian version was validated upon administration to 89 older patients previously categorised into healthy controls, mild cognitive impairment (MCI), and AD groups. The DAFS-BR could very accurately discriminate between healthy elderly controls and AD, as well as MCI^11^. The Direct Assessment of Functional Status-Revised (DAFS-R)^12^ was adapted and translated into Brazilian Portuguese in 2010^11^. However, over time, some tasks became outdated, indicating the need for a new cultural adaptation to contemporary Brazilian contexts, given the scale relevance for both clinical practice and research. In 2021, a group of researchers developed a second version, which was an adaptation of the first Brazilian version^13^. They changed some tasks for an update and included new tasks in two domains: communication: (1) sending e-mail and (2) sending messages on the WhatsApp^®^ application, and money-handling skills: (1) using a credit/debit card^13^. 

 Altogether, the lack of ADL measurement in DS (especially in Brazil), despite its usefulness for diagnostic purposes, tracking functioning decline over time, and planning interventions to enhance ADL independence in DS, makes the adaptation of the DAFS-BR for DS a worthy enterprise. This paper aims to describe the adaptation process of the DAFS to assess the functional capacity of adults with DS. 

## METHODS

### Study design and subjects

 This was a single-center, cross-sectional study conducted at Hospital das Clínicas Institute of Psychiatry, as part of a longitudinal study described by Conceição et al.^14^. Subjects with DS who were 20 years (or older), with or without suspected dementia, were recruited from among the patients being followed at the Aging and Down Syndrome Outpatient Clinic of the Institute of Psychiatry, where they were undergoing regular multidisciplinary assessments by physiotherapists, speech therapists, geriatricians, psychiatrists, otolaryngologists, neuropsychologists, occupational therapists, and dentists. Individuals with other mental or medical disorders that could affect cognition or functionality, such as depression and psychosis, were excluded (upon systematic psychiatric examination). All subjects had trisomy 21 confirmed by karyotype screening and most of them had simple trisomy. 

### Study design and ethics

 This was a single-center, cross-sectional study, carried out in the Laboratory of Neuroscience at Hospital das Clínicas Institute of Psychiatry (Faculty of Medicine, University of São Paulo), in the city of São Paulo, Brazil. The study was approved by the Research Ethics Committee of the Faculty of Medicine, University of São Paulo, and was registered at Plataforma Brasil, operated by the Brazilian National Research Ethics Committee (CAAE no. 40401420.7.0000.0068). All subjects or their legal guardians gave written informed consent. This adaptation was conducted by the same authors who translated and studied the first Brazilian version of DAFS (DAFS-BR)^11^. Therefore, no formal authorizations were necessary. 

### Pilot study: adapting the Brazilian-version scale for the Down syndrome population

 The adaptation process is a complex process that requires methodological rigour. Namely, adaptation refers to adjusting an instrument to assess a specific population, typically involving changes to terminology, content, and other culturally, psychologically, or linguistically relevant variables^15^. 

 The adaptation process for the target population involved administering the first DAFS-BR^11^ to a group of 15 adults with DS. For this, “Test Development Guidelines” were used, which suggest adaptations considering linguistic, psychological, and cultural idiosyncrasies in the target population. Specialists with relevant experience in each domain offered their inputs. After phase one, there was a discussion about the comprehensibility of each task with experts who proposed some changes. 

 Phase one of the pilot study was conducted by three occupational therapists, one of them being an expert in DS. This phase was very important because it used DAFS-BR in DS individuals, and new tasks considering the same original construct could be discussed and proposed after analysis of the performance of these subjects. According to Borsa,^16^ this phase of evaluation of the target population may be conducted once or multiple times, depending on the needs and complexity of the instrument being adapted. 

 Phase two involved experts in the DS population, including neuropsychologists, speech therapists, social workers, occupational therapists, psychiatrists, dentists, and physiotherapists, to evaluate their understanding of the vocabulary used in the instructions of the Direct Assessment of Functional Status for people with Down syndrome (DAFS-DS). This phase is commonly carried out only with the target population. However, it was implemented here with DS experts to ensure the accuracy and applicability of the adapted materials^17^. In this phase, the final version of the DAFS was administered to individuals with DS. 

## OBJECTIVE

 The present study aims to describe the adaptation process of the DAFS for Brazilian adults with DS. 

## RESULTS

### Phase one

 Firstly, the DAFS-BR was administered to 15 adults with DS (nine men and six women), divided into two diagnostic groups: stable cognition and suspected dementia or cognitive impairment. After that, three experts in a committee discussed and identified the modifications across domains and tasks that would be necessary. Changes were made in the time orientation, communication (telephone use), money-handling skills, and shopping skills domains. In the “communication” domain, telephone numbers originally containing seven digits were updated to eight digits. Furthermore, we have included the option of using a cell phone for the same task. Despite such adaptations, this item score remained unchanged. Adjustments were also made to the “time orientation” domain. The “telling time” task from a digital clock in Arabic numerals was included as a facultative alternative to the standard analogue clock. Also, in “date orientation”, we have included the option of showing a (current) calendar to the subjects. The total score for this task was maintained (Table 1 ). 

**Table 1:** Changes made to the tasks after the adaptation.

Assessed construct	DAFS-BR tasks	DAFS-SD tasks
Time orientation	1. Ability to read an **analogue** clock and tell the time.	1. Ability to read a **digital** clock and tell the time.
	2. Date orientation: day of the month, day of the week, month, and year.	2. Date orientation to the date: day of the month, day of the week, month, and year.
Communication	1. Use a **landline telephone** and prepare a letter to be mailed.	1. Use a **cell phone** and prepare a message to be sent.
	2. Telephone numbers originally containing seven digits	2. Telephone numbers were updated to eight digits
Money-handling skills	1. Identify current currency (bills and coins).	1. Identify current currency (bills and coins).
	2. Count current currency (mental calculation).	2. Count current currency (**using a calculator**).
	3. Check the change using mechanical calculation.	3. Check the change using a store receipt (**using a calculator**).
	4. Fill out a bank check.	**4. It was removed because this population does not use checks.**
	5. Calculate the balance of a bank account.	**5. It was removed because it is not common for this population to check bank balance.**
Shopping skills	1. Recall a list of **6 products** (memorized during the test).	1. Recall a list of **2 products** from the supermarket (memorized during the test).
	2. Recognize the **6 products** from the memorized list.	2. Recognize **4 products** from a list of pictures.
	3. Select supermarket items from a **written** list.	3. Select **4 products** from a **verbally provided** list.
Dressing and personal hygiene	1. Button up a shirt.	1. Button up a shirt.
	2. Zip up a shirt.	2. Zip up a shirt.
	3. Tie a shoelace.	3. Tie a shoelace.
	4. Wash hands with liquid soap.	4. Wash hands with liquid soap.
Eating	1. Use a knife and fork to cut meat.	1. Use a knife and fork to cut meat.
	2. Use a spoon to eat soup.	2. Use a spoon to eat soup.
	3. Pour water into a cup and drink.	3. Pour water into a cup and drink.
Traffic signs	Not included (removed in the translation and adaptation process by Pereira et al.)^11^ .	Recognize pedestrian traffic signs:
		1. Pedestrian signal – **green**.
		2. Pedestrian signal – **red** .
		3. **Crosswalk**.
Total score	106	108

 Finally, the task “preparing a letter” was replaced with the task “sending a message”, where patients had to send a WhatsApp ^®^ message - written or spoken – or simply write that message on a piece of paper. For illiterate individuals, however, the message could also be conveyed verbally to researchers. 

 Concerning the item “money-handling skills”, the currency was shifted to Brazilian Reais, whereas in “counting currency”, we changed calculation demands to simpler and easier ones. Conversely, in “making change”, we used a purchase receipt for each task that is identical to those used at commercial establishments in Brazil, showing change values (i.e., change is provided along with the receipt). The last task includes four trial conditions, two of which have the correct change and vice versa, and the subjects are required to discriminate between two correct and two incorrect trials. 

 As for the item “shopping skills”, the total score was maintained. Nonetheless, in the subdomain “shopping recall”, a reduction of two items to be memorised was suggested by experts. In addition, in the “shopping recall” task using a “written list”, it was also suggested that a list of pictures with the same items could be offered as an optional resource for illiterate subjects. Furthermore, for “shopping skills”, an additional task was also implemented: “shopping with verbal instructions”, where four items are verbally requested from the store. This task is important because it analyses an individual’s ability to follow verbal instructions. Of note, all tasks involving the domains “dressing and personal hygiene” and “eating” were maintained in full, including the scoring system. 

 Additionally, there was one more change related to walking in the streets independently. In the original American version of the DAFS-R^12^, the “traffic signs” domain, for drivers, was previously culturally adapted for the DAFS-BR^11^. However, for the present version of the scale, we used a task to assess the ability to recognise traffic signs from a pedestrian perspective. As such, we employed a new task to assess subjects’ ability to identify/discriminate between pictures with a “little green man light” (i.e., indicating that pedestrians may safely cross the street) from those with “a little man red light” (i.e., the pedestrian has to wait to cross the street), as well as the ability to recognise an actual crosswalk. 

 Finally, the first version of DAFS-DS was concluded. 

### Phase two

 Once the first version of DAFS-DS was concluded, a group of seven DS experts (one physiotherapist, two speech therapists, one neuropsychologist, one occupational therapist, one psychiatrist, and one dentist) discussed the following:

- (cultural and semantic) equivalences and,
- structure, layout, and instructions.

This phase was essential for verifying the applicability and comprehensibility of newly adapted tasks, considering the target population and clinicians. 

 The discussion ended when total agreement was reached for all items. 

 An important and last change made in this phase was the order of domains. It was decided to reverse the order of tasks to organize them in increasing order of complexity. First, basic ADLs and instrumental activities of daily living (more complex activities) became the last to be evaluated. 

 When this phase was complete, the second version of DAFS-DS was tested on all 15 DS subjects assessed previously. This version was proven to be successful, eliminating the need for further discussion among specialists. 

**Figure 1:**
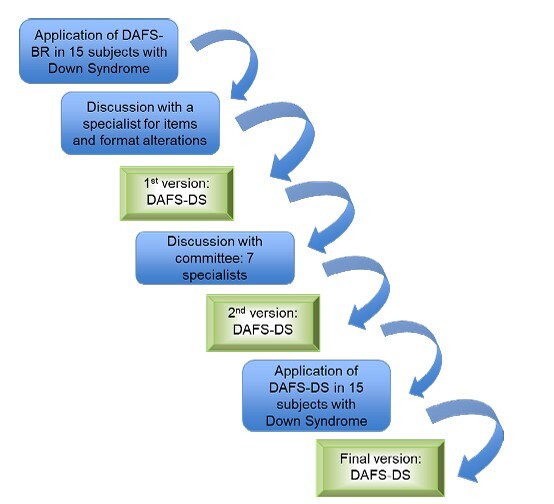
Flow chart of the adaptation process.

 The entire adaptation process is illustrated in Figure 1. 

## DISCUSSION

 This study involved an extensive process that included discussions among experts and the application of a test in the target population. 

 Changes were made in the tasks because DAFS-BR was developed considering the general population. Individuals with DS have intellectual disability characterized by impaired cognition, including executive function, working memory, and difficulty storing and processing verbal information such as sequences, instructions, and new vocabulary or information^18,19^. 

 As a limitation, there are no clear standards to adapt assessments for a specific population. Even in a recent systematic review of guidelines for assessment adaptation, no rules or information were found about adapting instruments to a specific population^20^. In this study, the pilot study had two phases. In phase one, DAFSBR was tested with the target population. According to Borsa et al.^16^, there is no consensus in the literature about the specific steps taken in this process. Therefore, evaluation can be conducted at any time, depending on the need and the complexity of the instrument to be adapted. Rather, the appropriateness of the items and the structure of the instrument was considered clear, appropriate, and well-written at the end of the process. 

 In conclusion, it is important to emphasize that this study was an adaptation according to the target population. No statistical procedures were performed. 

 The focus is on assessing the adequacy of the items and the structure of the instrument, whether the terms are clear, correspond to everyday reality, and are well formulated. When an item is not understood, for example, respondents could be asked to suggest synonyms that better represent the vocabulary of the group for whom the instrument is intended. 

 At present, we are conducting data collection for a validation and reliability study. This could contribute to the publication of a standardised functional assessment for rehabilitation professionals who work with DS individuals. Once psychometric studies have been performed, the DAFS-DS may be considered the first ecological instrument for occupational therapists and neuropsychologists to monitor functional decline in adults with DS in Brazil.^21^

## Data Availability

The datasets generated and/or analyzed during the current study are available from the corresponding author upon reasonable request.
